# Investigation of *Plasmodium falciparum* pfhrp2 and pfhrp3 gene deletions and performance of a rapid diagnostic test for identifying asymptomatic malaria infection in northern Ethiopia, 2015

**DOI:** 10.1186/s12936-022-04097-7

**Published:** 2022-03-04

**Authors:** Colleen M. Leonard, Ashenafi Assefa, Jessica N. McCaffery, Camelia Herman, Mateusz Plucinski, Heven Sime, Hussein Mohammed, Amha Kebede, Hiwot Solomon, Mebrahtom Haile, Matt Murphy, Jimee Hwang, Eric Rogier

**Affiliations:** 1 Malaria Branch, Division of Parasitic Diseases and Malaria, Centers for Disease Control and Prevention, Atlanta, GA 30329 USA; 2 Oak Ridge Institute for Science and Education, US. Department of Energy, Oak Ridge, TN 37831 USA; 3 Ethiopian Public Health Institute, Addis Ababa, Ethiopia; 4 Infectious Disease Ecology and Epidemiology Laboratory, University of North Carolina at Chapel Hill, Chapel Hill, USA; 5 U.S. President’s Malaria Initiative, Malaria Branch, Division of Parasitic Diseases and Malaria, Centers for Disease Control and Prevention, Atlanta, GA 30329 USA; 6 African Society for Laboratory Medicine, Addis Ababa, Ethiopia; 7 Ethiopian Federal Ministry of Health, Addis Ababa, Ethiopia

**Keywords:** Malaria, Antigenemia, HRP2, pLDH, RDT, HRP3, Gene deletions

## Abstract

**Background:**

Rapid diagnostic tests (RDTs) are widely used for malaria diagnosis of both symptomatic and asymptomatic infections. Although RDTs are a reliable and practical diagnostic tool, the sensitivity of histidine-rich protein 2 (HRP2)-based RDTs can be reduced if *pfhrp2* or *pfhrp3* (*pfhrp2/3*) gene deletions exist in the *Plasmodium falciparum* parasite population. This study evaluated dried blood spot (DBS) samples collected from a national household survey to investigate the presence of *pfhrp2/3* deletions and the performance of the RDT used in the cross-sectional survey in a low transmission setting.

**Methods:**

The 2015 Ethiopia Malaria Indicator Survey tested household members by RDT and collected DBS samples. DBS (n = 2648) from three regions in northern Ethiopia were tested by multiplex bead-based antigen detection assay after completion of the survey. The multiplex assay detected pan-*Plasmodium* lactate dehydrogenase (LDH), pAldolase, and HRP2 antigens in samples. Samples suspected for *pfhrp2/3* gene deletions (pLDH and/or pAldolase positive but low or absent HRP2) were further investigated by molecular assays for gene deletions. Antigen results were also compared to each individual’s RDT results. Dose–response logistic regression models were fit to estimate RDT level of detection (LOD) antigen concentrations at which 50, 75, 90, and 95% of the RDTs returned a positive result during this survey.

**Results:**

Out of 2,648 samples assayed, 29 were positive for pLDH or pAldolase antigens but low or absent for HRP2 signal, and 15 of these samples (51.7%) were successfully genotyped for *pfhrp2/3*. Of these 15 *P. falciparum* infections, eight showed single deletions in *pfhrp3,* one showed a single *pfhrp2* deletion, and six were *pfhrp2/3* double-deletions. Six *pfhrp2* deletions were observed in Tigray and one in Amhara. Twenty-five were positive for HRP2 by the survey RDT while the more sensitive bead assay detected 30 HRP2-positive samples. A lower concentration of HRP2 antigen generated a positive test result by RDT compared to pLDH (95% LOD: 16.9 ng/mL vs. 319.2 ng/mL, respectively).

**Conclusions:**

There is evidence of dual *pfhrp2/3* gene deletions in the Tigray and Amhara regions of Ethiopia in 2015. As the prevalence of malaria was very low (< 2%), it is difficult to make strong conclusions on RDT performance, but these results challenge the utility of biomarkers in household surveys in very low transmission settings.

**Supplementary Information:**

The online version contains supplementary material available at 10.1186/s12936-022-04097-7.

## Background

Malaria rapid diagnostic tests (RDTs) are a reliable and practical tool for identifying malaria infection, particularly in resource-limited settings where microscopy expertise may be limited [1, 2]. RDTs do not require electricity and typically return results in less than 30 min [1]. In many African countries, RDTs are the primary method used for parasitological confirmation of malaria illness [3]. Malaria RDTs detect one or more malaria antigens [1] with the majority of the RDTs used in sub-Saharan Africa detecting only the *Plasmodium falciparum* histidine-rich protein 2 (HRP2) antigen due to the predominance of *P. falciparum* malaria in this region [3]. Other RDT targets are also available to detect other *Plasmodium* antigens, such as pan-*Plasmodium* epitopes for lactate dehydrogenase (pLDH), *P. falciparum* LDH, and *Plasmodium vivax* LDH.

Although RDTs are a reliable and practical diagnostic tool, they are not without limitations. Notably, the accuracy of HRP2-based RDTs may be limited in the presence of high prevalence of *pfhrp2* and *pfhrp3* (*pfhrp2/3*) gene deletions in the parasite population [4]. *Pfhrp2* gene deletions render the parasite unable to produce HRP2 antigens and, therefore, reduce the accuracy of HRP2-based RDTs for detection of *P. falciparum* infection. The closely-related HRP3 antigen can also be detected by HRP2-based RDTs due to the presence of similar repeat epitopes targeted by the anti-HRP2 diagnostic antibodies although with lower sensitivity [5]. Nonetheless, a high prevalence of *pfhrp2* or dual *pfhrp2* and *pfhrp3* gene deletions pose a threat to the identification of *P. falciparum* infection if only utilizing HRP2-based RDTs for diagnostic confirmation. The World Health Organization (WHO) recommends that a 5% or greater local prevalence of *pfhrp2* deletions among symptomatic persons causing false negative HRP2 RDT results warrants a change in RDT type to a non-HRP2 based RDT [6].

Throughout Africa, *pfhrp2/3* deletions have generally been found to be rare (< 5% prevalence) [7]. However, a high prevalence of *pfhrp2/3* gene deletions has been reported in parts of East Africa. In 2016, a 62% prevalence of *pfhrp2* deletions was identified among *P. falciparum-*infected patients in Eritrea, which neighbours Ethiopia [8]. Additionally, of 79 PCR-confirmed *P. falciparum* samples collected from Djiboutian patients with suspected malaria in 2019, 87% had both *pfhrp2* and *pfhrp3* genes deleted [9]. Within Ethiopia, *pfhrp2/3* deletions have been reported in multiple regions, including Tigray, Amhara, Oromia, and Benishangul-Gumuz Regions [10–12]. The highest prevalence estimate of *pfhrp2*-deleted parasites causing false-negative RDT results published to date was in the Tigray Region (15%), which shares borders with Eritrea and Sudan [12].

Ethiopia has made great progress in moving toward malaria elimination with a national goal of elimination by the year 2030 [13]. In 2015, nationwide malaria prevalence in Ethiopia was estimated to be 1.2% by RDT [14]. Routine malaria indicator surveys (MIS) are conducted every 4–5 years in Ethiopia and enroll persons at their place of residence. The MIS utilizes both microscopy and RDTs to estimate the prevalence of malaria in the populace, with malaria treatment decisions made on the basis of RDT results [14]. Multispecies RDTs detecting HRP2 and pan-*Plasmodium* antigens were used in the Ethiopia MIS [14]. In very low transmission settings, there may be a high rate of false positive RDTs, which is why the U.S. President’s Malaria Initiative does not recommend collection of parasite burden by microscopy or RDT in national surveys in countries with < 3% malaria prevalence among children less than five [15].

The primary purpose of this research was to identify if participants in the Ethiopia MIS were infected with *pfhrp2/3* deleted *P. falciparum* parasites in 2015 as multiple reports have shown the presence of *P. falciparum* isolates with deletions of *pfhrp2* and/or *pfhrp3* genes in Ethiopia recently [10–12]. A multiplex bead-based antigen detection assay was utilized to rapidly screen the large number of dried blood spot samples collected during the 2015 Ethiopia MIS for potential *P. falciparum* HRP2/3 gene deletions (samples with low or no HRP2 and presence of pan-*Plasmodium* LDH antigens) [16]. A secondary objective was to use the already collected multiplex antigen assay results to assess the performance of the HRP2/pan-LDH RDT used during the MIS. While a comparison between RDT and bead antigen assay is not routinely recommended, it is useful to compare the RDT to a more sensitive diagnostic test to better characterize the parasite reservoir in this very low-prevalence setting. In addition, Ethiopia uses RDT prevalence estimates for strategic planning of malaria elimination. Other studies have assessed RDT performance in Ethiopia using microscopy or PCR as the “gold-standard” comparison [17–19]; however, microscopy and PCR both detect non-protein components of the *P. falciparum* parasite whereas the multiplex assay detects the same antigens as the RDT.

## Methods

### Study design and area

The Ethiopia MIS was a nationwide household survey conducted in 2015 from September 30 to December 10 and coincided with the high malaria transmission season [14]. The study used a two-stage cluster sampling methodology to select 555 enumeration areas proportional to population size and 25 households randomly within each area. Blood samples were collected for malaria testing from all children under five in every household and persons of all ages in every fourth household. Of the 2878 DBS samples available from the Amhara, Afar, and Tigray Regions in northern Ethiopia, RDT and bead assay results were generated for 2648 (92.0%) samples.

### Rapid diagnostic tests

Whole capillary blood samples were tested using the Carestart™ Pf/Pan RDT (AccessBio, Somerset NJ, U.S.), testing for the presence of HRP2 and pLDH antigens. Presence of HRP2 indicates *P. falciparum* infection, while a positive pLDH test indicates *P. falciparum* and/or another *Plasmodium* species. Participants with a positive RDT result were immediately treated for malaria according to the national malaria treatment guidelines [14].

### Multiplex bead-based antigen detection assay

DBS samples were shipped to the Malaria Laboratory at the Centers for Disease Control and Prevention in Atlanta, USA under ambient temperature for the retrospective analysis of *Plasmodium* antigens. Reagent preparation and the multiplex assay were performed as described previously [20]. For assay samples, a 6-mm punch equivalent of 10 µL whole blood was obtained from each DBS sample and blood eluted to a 1:20 concentration in blocking buffer (Buffer B: Phosphate Buffered Saline (PBS) containing 0.5% Bovine Serum Albumin (BSA), 0.05% Tween 20, 0.02% sodium azide, 0.5% polyvinyl alcohol, 0.8% polyvinylpyrrolidone and 3 µg/mL w/v *Escherichia coli* extract) overnight. The 1:20 whole blood dilution was used to detect HRP2, pLDH, and pAldolase antigens, and was run with samples in singlet on a MAGPIX machine with a target of 50 beads/region for each antigen. There is no reliable conversion between antigenaemia and parasitaemia. There is a positive correlation between the two values; however, HRP2 concentration can vary between individual infections with the same parasite density depending on the parasite’s production of HRP2 and the individual’s clearance of HRP2.

### *Plasmodium* species identification by PCR and *pfhrp2* and *pfhrp3* genotyping

DBS samples showing an atypically low signal for HRP2 antigen (compared to pLDH or pAldolase) [21, 22] were selected for DNA extraction (DNA Mini Kit, Qiagen, Valencia, CA) and *Plasmodium* species-specific photo-induced electron transfer (PET)-PCR and quantification of DNA [23]. Samples positive for *P. falciparum* DNA were subjected to nested PCR (nPCR) for the single-copy *pfmsp1* and *pfmsp2* genes as quality control for DNA quantity and integrity [24]. Only the samples for which both of these control genes were amplified were selected for further processing to determine the presence or absence of *pfhrp2* and *pfhrp3* genes. Genotyping for *pfhrp2* was performed by a single-step PCR amplifying a large section of *pfhrp2* and which was inclusive of both exons [25], and genotyping for *pfhrp3* was done through two nPCR reactions with primers specific for exon 1–2 and exon 2 regions [24]. All reactions were performed in singlet on two separate days, and for each PCR assay plate, a no template control (NTC), wild-type (3D7 strain), *pfhrp2* deleted (Dd2 strain), and *pfhrp3* deleted (HB3 strain) were all run in duplicate as controls. If amplification results were discordant, a third nPCR was performed as a tiebreaker.

### Statistical analysis

The minimum assay signal denoting positivity to *Plasmodium* antigens was determined by assaying a panel of 86 whole blood samples from US residents at 1:20 dilution. The lognormal mean + 3 standard deviations of the MFI-bg values for this sample set were used to calculate thresholds for each antigen target. To determine the performance of the Pf/Pan RDT based on antigen presence, sensitivity, specificity, and predictive values were calculated. Cohen’s kappa coefficient was calculated to determine the agreement between the RDT and bead-based antigen detection assay. To model for the association of RDT band result at given blood antigen concentrations, a non-parametric LOESS curve was generated to characterize the relationship between the likelihood of a positive RDT in the field and the log HRP2 or log pLDH concentration measured in the laboratory. A logistic regression model was fit to the dose–response data and was used to estimate the pLDH and HRP2 concentrations with 95% confidence intervals at which 50, 75, 90, and 95% of the RDTs would be expected to turn positive (level of detection [LOD]) [26]. All statistical analyses were completed in either SAS version 9.4 (SAS Institute, Cary, NC) or R version 4.03.3 (R Foundation for Statistical Computing, Vienna, Austria).

## Results

**Fig. 1:**
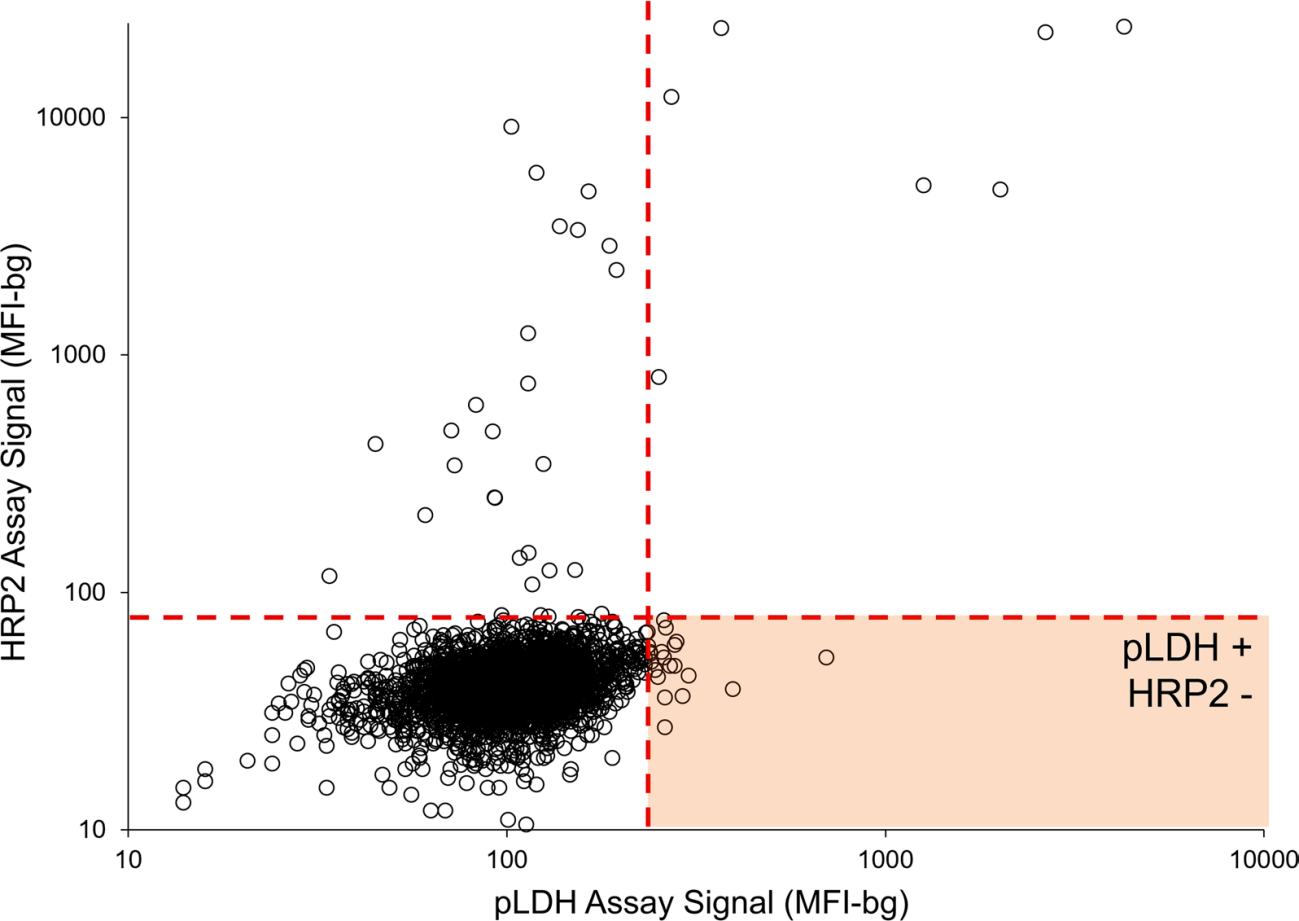
Selection of samples for *pfhrp2* and *pfhrp3* genotyping by ratio of LDH to HRP2. Vertical hashed red line designates the threshold for antigen positivity signal for pLDH antigen and the horizontal hashed red line designates the threshold for antigen positivity signal for HRP2. The region shaded in orange denotes values where DBS samples were positive for pLDH antigen, but negative for HRP2 antigen

Of the 2648 samples, about half (45.9%) were collected from children under 5 years of age, and slightly over half (51.5%) were from female participants (Table 1). For all samples, antigen assay signal as measured by the multiplex bead assay for pLDH or pAldolase was compared to HRP2 to identify samples with low or absent levels of HRP2 but positive for the other pan-*Plasmodium* antigens, which were then screened for potential *pfhrp2/3* deletions. The majority of specimens positive for either *Plasmodium* antigen had a high positive signal for HRP2, though a small subset of samples (n = 29) were positive for pLDH or pAldolase and had a low (or no) HRP2 signal (Fig. 1, Additional file 1).

**Table 1:** Characteristics of the study participants from the Amhara, Afar, and Tigray regions of Ethiopia, 2015

Characteristics	n (%)
Sex	
Female	1364 (51.5)
Male	1284 (48.5)
Age in years	
< 5	1215 (45.9)
5–14	473 (17.9)
15–24	312 (11.8)
≥ 25	648 (24.5)
RDT positive (any malaria species)	37 (1.40)
pLDH band positive by RDT	18 (0.68)
HRP2 band positive by RDT	25 (0.94)
Bead assay positive (for pLDH or HRP2)	35 (1.32)
pLDH positive by bead assay	13 (0.49)
HRP2 positive by bead assay	30 (1.1)

**Fig. 2:**
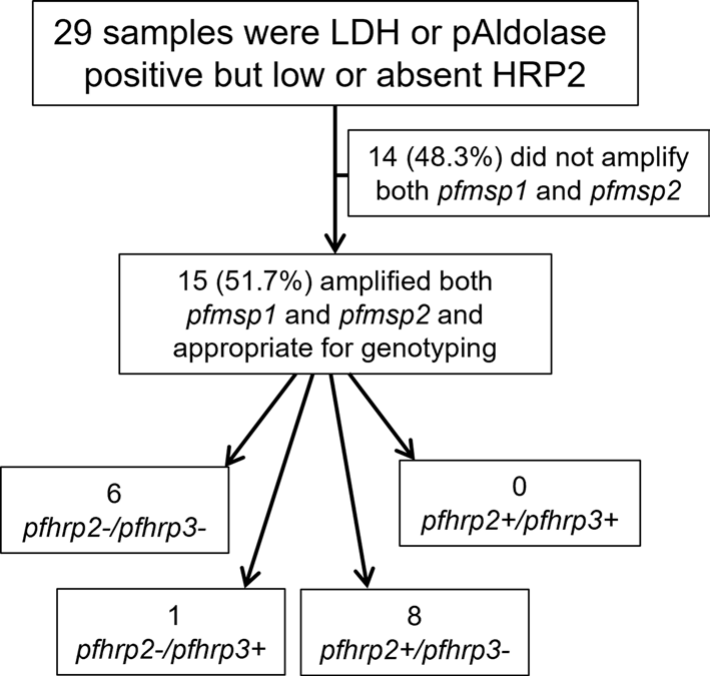
Genotyping results for *pfhrp2* and *pfhrp3* for samples LDH-positive but negative or low HRP2 antigens

**Fig. 3:**
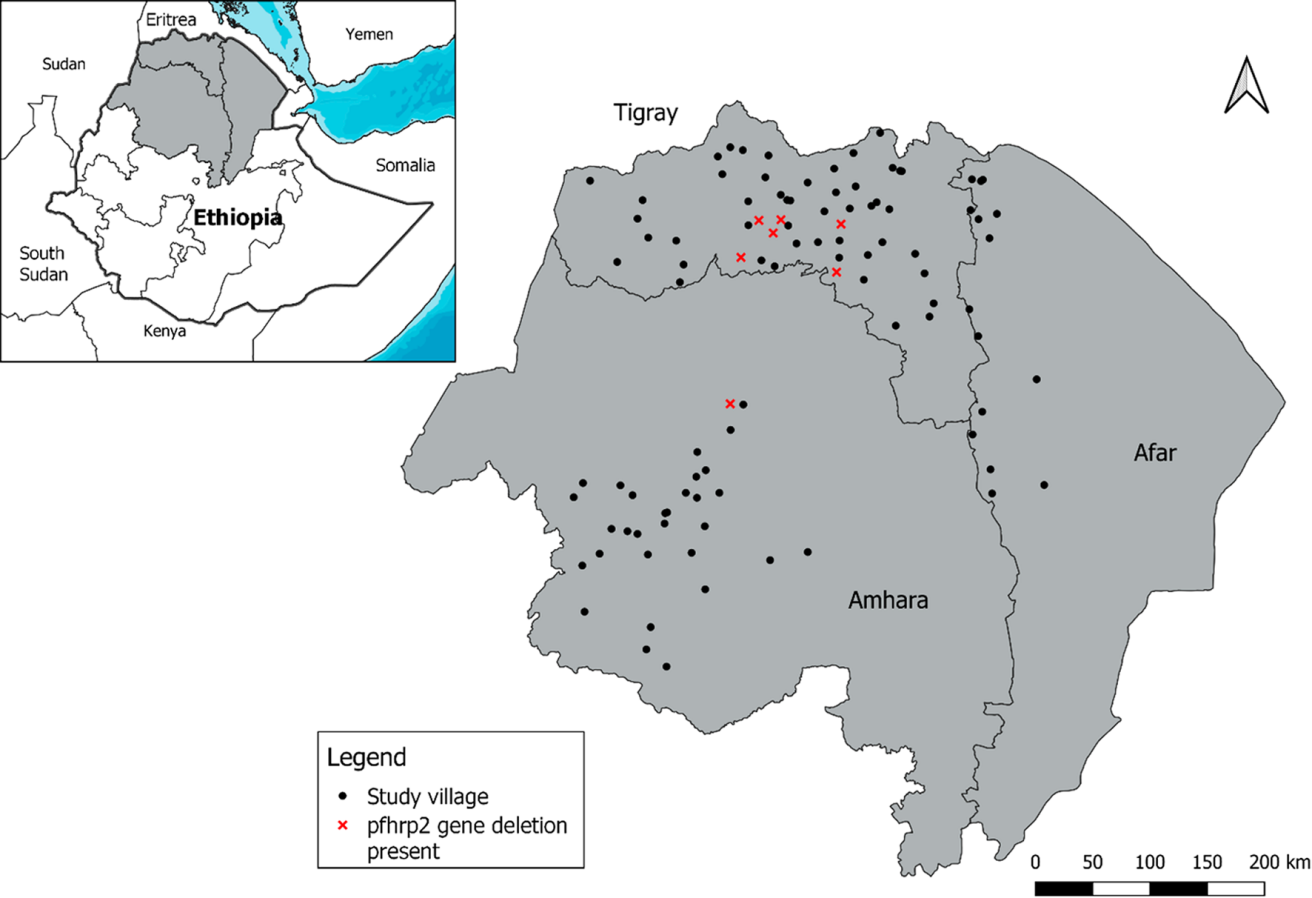
Location of *pfhrp2* gene deletions in the study regions. Identified *pfhrp2* gene deletions are shown at the village level by a red X, unless more than one gene deletion is present in which case locations were mapped with a “jitter” of 10–12 km to allow visualization of the multiple points

Samples with antigen profiles showing low (or no) HRP2 but high pLDH or pAldoase had DNA extracted from DBS and tested by PCR to confirm presence or absence of *P. falciparum* followed by further genotyping for the *pfhrp2/3* genes. From these 29 samples selected, 14 (48.3%) were unable to amplify the *pfmsp1* and *pfmsp2* single-copy genes and therefore could not have *pfhrp2/3* genotype reported (Fig. 2). Of these 14, 13 did not amplify *P. falciparum* 18S DNA by PET-PCR, and all 14 samples did not amplify *P. vivax*, *Plasmodium ovale,* or *Plasmodium malariae* DNA by PET-PCR. Of the 15 samples for which both *pfmsp1* and *pfmsp2* were amplified, eight had a genotype of *pfhrp2*+*/pfhrp3−*, one a genotype of *pfhrp2−/pfhrp3*+, and six a double-deleted genotype of *pfhrp2−/pfhrp3−.* None of the 15 samples were wild-type (*pfhrp2*+*/pfhrp3*+). Of the seven samples with deletions in the *pfhrp2* gene (the primary target of HRP2-based RDTs), six were found in the Tigray region and one in Amhara (Fig. 3).

From the 2648 DBS screened by the multiplex bead assay, the bead assay detected the pLDH antigen in 13 samples (0.49%), while 18 samples (0.68%) had a positive pLDH band result by RDT (Table 2). The bead assay detected HRP2 in 30 samples (1.1%) while 25 samples (0.94%) had a positive HRP2 band result by RDT. Using the bead assay as the gold-standard, the ability of the RDT to provide positive HRP2 and pLDH band results was retrospectively assessed for this 2015 MIS (Table 3). For a positive pLDH band result when LDH was present in the blood, the RDT exhibited a sensitivity and specificity of 53.8% (95% CI 25.1–80.8) and 99.6% (95% CI 99.3–99.8), respectively. The positive predictive value (PPV) and negative predictive value (NPV) for pLDH detection by RDT was 38.9% (95% CI 17.3–64.3) and 99.8% (99.6–99.9), respectively. For HRP2, the RDT exhibited a sensitivity and specificity of 53.3% (95% CI 34.3–71.7) and 99.7% (95% CI 99.3–99.8), respectively. The PPV and NPV for HRP2 detection by RDT was 64.0% (95% CI 42.5–82.0) and 99.5% (95% CI 99.1–99.7), respectively. There was moderate agreement between both tests for detecting pLDH and HRP2 antigens (Kappa = 0.45, Kappa = 0.58, respectively).

**Table 2:** RDT band positivity against the multiplex bead antigen assay for pLDH and HRP2

A			
		Bead assay pLDH	
		+	−
RDT	+	7	11
	−	6	2624

B			
		Bead assay HRP2	
		+	−
RDT	+	16	9
	−	14	2609

**Table 3:** RDT accuracy for detecting pLDH and HRP2 antigens compared to bead-based assay

		pLDH RDT band	HRP2 RDT band
Sensitivity	% (95% CI) n/N	53.8 (25.1–80.8) 7/13	53.3 (34.3–71.7) 16/30
Specificity	% (95% CI) n/N	99.6 (99.3–99.8) 2624/2635	99.7 (99.3–99.8) 2609/2618
PPV	% (95% CI)	38.9 (17.3–64.3)	64.0 (42.5–82.0)
NPV	% (95% CI)	99.8 (99.5–99.9)	99.5 (99.1–99.7)
Kappa coefficient	% (95% CI)	0.45 (0.23–0.67)	0.58 (0.42–0.73)

CI: Confidence interval; PPV: Positive predictive value; NPV: Negative predictive value

**Fig. 4:**
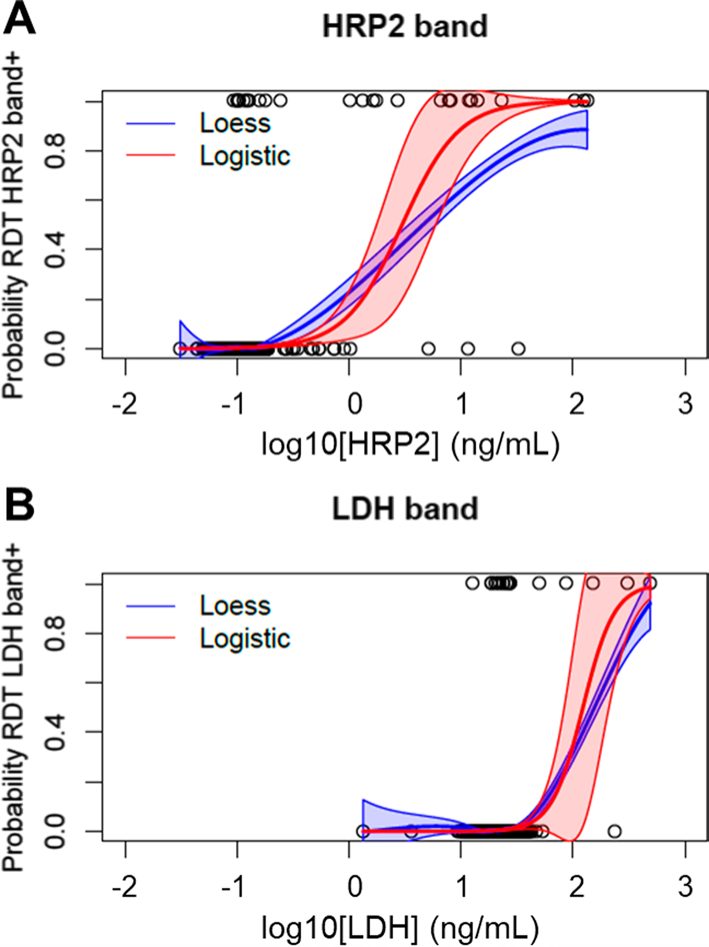
Dose–response modeling for probability of RDT band positivity as a factor of blood antigen concentration. Antigen concentration, log_10_[HRP2] (**A**) and log_10_[LDH] (**B**), in ng/mL versus binary rapid diagnostic test result for the HRP2 band (**A**) and LDH band (**B**). The light red region surrounding the red line represents the 95% confidence interval (CI) for the logistic regression curve. The light blue region surrounding the blue line represents the 95% CI for the Loess regression curve

Assessment of RDT result by antigen concentration in blood found detection of HRP2 to be more sensitive than detection of the pLDH antigen. Both LOESS and logistic models showed a clear sigmoidal shape of higher antigen levels being more predictive of a positive band reading (Fig. 4). For RDTs performed in Tigray, Amhara, and Afar during the 2015 MIS, dose–response logistic modeling estimated that an HRP2 blood concentration of 3.0 ng/mL would elicit a positive HRP2 band result 50% of the time, whereas a pLDH concentration of 123.2 ng/mL would elicit a positive pLDH band result 50% of the time (Table 4). If increasing the sensitivity of a positive result to 95%, these estimates were 16.9 ng/mL for the HRP2 band and 319.2 ng/mL for the pLDH band.

**Table 4:** LOESS and logistic regression estimates by sensitivity of positive RDT band result

Antigen and model	Sensitivity level			
	50% ng/mL (95% CI)	75% ng/mL (95% CI)	90% ng/mL (95% CI)	95% ng/mL (95% CI)
HRP2				
LOESS	4.6 (3.4–6)	21 (16–29)	NA (53–NA)	NA (101–NA)
Logistic	3.0 (1.8–6)	5.7 (2.7–11)	10.9 (3.7–21)	16.9 (4.2–34)
pLDH				
LOESS	156 (142–174)	289 (249–359)	455 (353–NA)	NA (400–NA)
Logistic	123.2 (84–211)	175.6 (102–287)	250.5 (115–409)	319.2 (120–NA)

## Discussion

This investigation sought to identify the presence of deletions in genes that code for *P. falciparum* HRP2 and HRP3 (RDT antigen targets) in DBS samples from northern Ethiopia collected as part of the Ethiopia MIS in 2015. A highly-sensitive laboratory assay was utilized to detect antigenemia, and used the antigen results to select samples for molecular genotyping. The current study found evidence for deletion in the *pfhrp2* and *pfhrp3* genes of *P. falciparum* samples from northern Ethiopia obtained in 2015—mainly in the Tigray Region. Of the 15 samples successfully genotyped, six showed *pfhrp2/3* double-deletions, eight showed single *pfhrp3* deletions, and one showed a single *pfhrp2* deletion. Six of the seven samples with *pfhrp2* deletions were observed in Tigray. Since *pfhrp2/3* double-deleted parasites do not produce the HRP2/HRP3 antigen targets of the RDT, assessment of these parasites with HRP-based RDTs produces a high false-negative rate [27]. False-negative RDT results not only prevent appropriate treatment when needed but could also impede malaria control and elimination efforts by systematically under-reporting true prevalence. Combination RDTs using HRP2/pLDH or HRP2/PfLDH detection may be better suited for detecting *pfhrp2*-deleted parasites, though sensitivity may be compromised [27]. One study found that pLDH-only RDTs detected 95.6% of all *pfhrp2*-deleted parasites, which provided a much more sensitive alternative to HRP2-only, Pf-LDH, or HRP2-combination RDTs [27]. The study design for genotyping sample selection here was not intended to provide exact prevalence estimates, but rather to investigate the presence of these deletions, and if any geographical clustering was noted. *Pfhrp2/3* gene deletions have been previously reported in the Oromia and Benishangul-Gumuz Regions of Ethiopia from cross-sectional health facility-based studies in the years 2015 and 2018, respectively [10, 11].

Furthermore, a multi-site study conducted according to WHO’s protocol on *pfhrp2/3* deletion surveillance found evidence of high prevalence (9.7%) of *pfhrp2* gene deletions in the Amhara and Tigray regions, specifically in areas bordering Sudan and Eritrea (2017–2018 WHO survey) [12]. This report adds to the growing literature that *pfhrp2/3* deletions are of concern in Ethiopia [10–12], and more extensive surveys which are underway will help facilitate decisions regarding use of appropriate and effective RDTs in Ethiopia.

With the already-collected antigen data, we also investigated HRP2/pLDH RDT performance in the context of a cross-sectional survey in a low transmission setting. When treating the bead assay as the gold-standard, the specificity of the RDT for detecting pLDH and HRP2 antigens was very high (99.6% and 99.7%, respectively); however, low sensitivity was found for both pLDH and HRP2 antigens (53.8% and 53.3%, respectively). Reduced sensitivity of malaria RDTs in detecting asymptomatic infections during community surveys has been documented previously [28]. This association is likely seen due to the increased observation of low-parasite density infections obtained from mostly asymptomatic persons, especially in low-transmission settings. Also, multiclonal infections (*pfhrp2/3*-deleted clones) may cause the *Plasmodium* parasite to evade detection by the HRP2-based RDTs. This finding is not surprising given that RDTs were originally designed to detect symptomatic malaria infections (about > 500 parasites/µL) and not low-density, asymptomatic infections [28, 29]. The similar sensitivities for pLDH and HRP2 antigens found in this study contrasts with the findings from other studies, which have shown a higher sensitivity for HRP2 compared to pLDH antigens [5, 13]. There were 11 false positives for detecting the pLDH RDT band and 9 for the HRP2 band. This resulted in a low PPV for detecting both antigens by RDT; however, the PPV for a positive HRP2 RDT was higher than for the pLDH antigen (64.0% vs. 38.9%). For pLDH, the RDT detected slightly more samples compared to the bead assay (18 vs. 13 samples). These may have been false positive RDT results, or the bead assay may not have detected pLDH due to various reasons, such as the quality of the DBS sample received, or the DBS may have led to antigen degradation of the sample. The prevalence of malaria by RDT was similar to the prevalence by bead assay from this Ethiopian household survey; however, this may be due to the small number of positive samples by either test and the similar number of false positives and false negatives. In low-transmission settings, RDTs may generate many false negative results since they cannot reliably detect low-density parasite infections [30]. Additionally, false positive results are common in low-transmission settings due to the low background prevalence [31]. Therefore, RDT prevalence estimates can be a less reliable indicator of true malaria burden in a population in low transmission settings. These findings of low sensitivity and PPV support recommendations to not collect biomarkers through national surveys in settings of very low prevalence.

The RDT detected a wide range of HRP2 and pLDH antigen concentrations, but a lower concentration of HRP2 antigens was required to elicit a positive RDT response compared to the LDH antigen. The lowest reliably detected (95% LOD) HRP2 concentration found here (16.9 ng/mL) was within the range found by other studies, 1.5–109 ng/mL [26, 32]. The RDT LOD seems to vary based on the target population and endemic setting. Wide variation in HRP2-detecting RDT sensitivity was found in one study which assessed RDT performance from multiple field surveys across four countries and included community and health facility-based samples [26].

This study has several important limitations. The samples used were collected from a cross-sectional community-based survey in 2015 and therefore, the study design was not intended to provide exact *pfhrp2/3* deletion prevalence estimates according to the WHO protocol [33]. Because only *P. falciparum* samples with no or low HRP2 signal were assessed for *pfhrp2/3* deletions, this study may have under- or over- estimated the *pfhrp2/3* deletions. Lingering HRP2 antigen from a previous infection may have caused us to miss some samples with current *pfhrp2* deletions. Also, there was a low number of malaria positive specimens (less than 40 samples positive by RDT) to assess the performance of the RDT and less than 50 samples positive by bead assay for pLDH, pAldolase or HRP2 antigens to assess the presence of *pfhrp2/3* deletions. The number of false negative and false positive samples for pLDH (6 and 11) and HRP2 (14 and 9) were also small. Additionally, a low parasite density infection (common among asymptomatic persons) may not provide a high-quality sample for detection of gene deletions by PCR. For this reason, the WHO recommends assessing *pfhrp2/3* gene deletions among symptomatic persons [6]. Conventional PCR assays were utilized in this study, but recently, multiplexed quantitative PCR assays offer pragmatic testing workflows for these *pfhrp2/3* deletions [34].

## Conclusions

Using samples collected from a national malaria survey, this study reports the presence of *pfhrp2/3-*deleted *P. falciparum* parasites in the Tigray and Amhara regions of Ethiopia in 2015. Follow-up surveys in northern and other parts of Ethiopia should attempt to generate prevalence estimates for *pfhrp2/3* deletions and pilot non-HRP2 RDTs. Also, assessment of the HRP2 and LDH antigen concentrations was able to provide a more detailed characterization of RDT performance in this field setting and found the sensitivity and PPV of the RDT was low in this 2015 household survey. As the prevalence of malaria was very low in this population (< 2%), it is difficult to make strong conclusions on the RDT performance, but does call into question the utility of biomarkers in household surveys in very low transmission settings.

## Supplementary Information

**Additional file 1.** Selection of samples for *pfhrp2* and *pfhrp3* genotyping by ratio of pAldolase to HRP2.The vertical hashed red line designates the threshold for antigen positivity signal for pAldolase antigen and the horizontal hashed red line designates the threshold for antigen positivity signal for HRP2. The region shaded in orange denotes values where DBS samples were positive for pAldolase antigen, but negative for HRP2 antigen.

## Data Availability

Data is available upon reasonable request to the corresponding author and approval from the Ethiopian Public Health Institute.

## References

[1] Moody A (2002). Rapid diagnostic tests for malaria parasites. Clin Microbiol Rev.

[2] Rosenthal PJ (2012). How do we best diagnose malaria in Africa?. Am J Trop Med Hyg.

[3] WHO (2019). World malaria report 2019.

[4] Cheng Q, Gatton ML, Barnwell J, Chiodini P, McCarthy J, Bell D (2014). *Plasmodium falciparum* parasites lacking histidine-rich protein 2 and 3: a review and recommendations for accurate reporting. Malar J.

[5] Poti KE, Sullivan DJ, Dondorp AM, Woodrow CJ (2020). HRP2: transforming malaria diagnosis, but with caveats. Trends Parasitol.

[6] WHO Global Malaria Programme. False-negative RDT results and *P. falciparum* histidine-rich protein 2/3 gene deletions. Geneva: World Health Organization; 2016. https://www.who.int/publications/i/item/WHO-HTM-GMP-2017.18.

[7] Thomson R, Parr JB, Cheng Q, Chenet S, Perkins M, Cunningham J (2020). Prevalence of *Plasmodium falciparum* lacking histidine-rich proteins 2 and 3: a systematic review. Bull World Health Organ.

[8] Berhane A, Anderson K, Mihreteab S, Gresty K, Rogier E, Mohamed S (2018). Major threat to malaria control programs by *Plasmodium falciparum* lacking histidine-rich protein 2 Eritrea. Emerg Infect Dis.

[9] Iriart X, Menard S, Chauvin P, Mohamed HS, Charpentier E, Mohamed MA (2020). Misdiagnosis of imported falciparum malaria from African areas due to an increased prevalence of pfhrp2/pfhrp3 gene deletion: the Djibouti case. Emerg Microbes Infect.

[10] Alemayehu GS, Blackburn K, Lopez K, Cambel Dieng C, Lo E, Janies D (2021). Detection of high prevalence of *Plasmodium falciparum* histidine-rich protein 2/3 gene deletions in Assosa zone, Ethiopia: implication for malaria diagnosis. Malar J.

[11] Golassa L, Messele A, Amambua-Ngwa A, Swedberg G (2020). High prevalence and extended deletions in *Plasmodium falciparum* hrp2/3 genomic loci in Ethiopia. PLoS ONE.

[12] Feleke SM, Reichert EN, Mohammed H, Brhane BG, Mekete K, Mamo H (2021). *Plasmodium falciparum* is evolving to escape malaria rapid diagnostic tests in Ethiopia. Nat Microbiol.

[13] Ethiopia Ministry of Health. Ethiopia malaria elimination strategic plan: 2021–2025. 2020. https://e-library.moh.gov.et/library/wp-content/uploads/2021/06/Ethiopia-Malaria-Elimination-Strategic-Plan-2021-2025-Agust-31.pdf Accessed 9 Jul 2021.

[14] Ethiopian Public Health Institute. National Malaria Indicator Survey, 2015. 2016. https://www.ephi.gov.et/images/pictures/download2009/MIS-2015-Final-Report-December-_2016.pdf. Accessed 07 Jul 2021.

[15] U.S. President's Malaria Initiative. U.S. President's Malaria Initiative FY 2020 Guidance. 2019. https://d1u4sg1s9ptc4z.cloudfront.net/uploads/2021/03/pmi-technical-guidance-1.pdf. Accessed 27 Jan 2022.

[16] Rogier E, Plucinski M, Lucchi N, Mace K, Chang M, Lemoine JF (2017). Bead-based immunoassay allows sub-picogram detection of histidine-rich protein 2 from *Plasmodium falciparum* and estimates reliability of malaria rapid diagnostic tests. PLoS ONE.

[17] Getnet G, Getie S, Srivastava M, Birhan W, Fola AA, Noedl H (2015). Diagnostic performance of rapid diagnostic tests for the diagnosis of malaria at public health facilities in north-west Ethiopia. Trop Med Int Health.

[18] Ashton RA, Kefyalew T, Tesfaye G, Counihan H, Yadeta D, Cundill B (2010). Performance of three multi-species rapid diagnostic tests for diagnosis of *Plasmodium falciparum* and *Plasmodium vivax* malaria in Oromia Regional State, Ethiopia. Malar J.

[19] Eticha T, Tamire T, Bati T (2020). Performance evaluation of malaria Pf/Pv combo test kit at highly malaria-endemic area, Southern Ethiopia: a cross-sectional study. J Trop Med.

[20] Plucinski MM, Herman C, Jones S, Dimbu R, Fortes F, Ljolje D (2019). Screening for Pfhrp2/3-deleted *Plasmodium falciparum*, non-falciparum, and low-density malaria infections by a multiplex antigen assay. J Infect Dis.

[21] Bakari C, Jones S, Subramaniam G, Mandara CI, Chiduo MG, Rumisha S (2020). Community-based surveys for *Plasmodium falciparum* pfhrp2 and pfhrp3 gene deletions in selected regions of mainland Tanzania. Malar J.

[22] Plucinski MM, Candrinho B, Dimene M, Colborn J, Lu A, Nace D (2019). Assessing performance of HRP2 antigen detection for malaria diagnosis in Mozambique. J Clin Microbiol.

[23] Lucchi NW, Narayanan J, Karell MA, Xayavong M, Kariuki S, DaSilva AJ (2013). Molecular diagnosis of malaria by photo-induced electron transfer fluorogenic primers: PET-PCR. PLoS ONE.

[24] Abdallah JF, Okoth SA, Fontecha GA, Torres RE, Banegas EI, Matute ML (2015). Prevalence of pfhrp2 and pfhrp3 gene deletions in Puerto Lempira, Honduras. Malar J.

[25] Jones S, Subramaniam G, Plucinski MM, Patel D, Padilla J, Aidoo M (2020). One-step PCR: a novel protocol for determination of pfhrp2 deletion status in *Plasmodium falciparum*. PLoS ONE.

[26] Plucinski M, Dimbu R, Candrinho B, Colborn J, Badiane A, Ndiaye D (2017). Malaria surveys using rapid diagnostic tests and validation of results using post hoc quantification of *Plasmodium falciparum* histidine-rich protein 2. Malar J.

[27] Gatton ML, Chaudhry A, Glenn J, Wilson S, Ah Y, Kong A (2020). Impact of *Plasmodium falciparum* gene deletions on malaria rapid diagnostic test performance. Malar J.

[28] Watson OJ, Sumner KM, Janko M, Goel V, Winskill P, Slater HC (2019). False-negative malaria rapid diagnostic test results and their impact on community-based malaria surveys in sub-Saharan Africa. BMJ Glob Health.

[29] Slater HC, Ross A, Felger I, Hofmann NE, Robinson L, Cook J (2019). The temporal dynamics and infectiousness of subpatent *Plasmodium falciparum* infections in relation to parasite density. Nat Commun.

[30] McMorrow ML, Aidoo M, Kachur SP (2011). Malaria rapid diagnostic tests in elimination settings–can they find the last parasite?. Clin Microbiol Infect.

[31] Ranadive N, Kunene S, Darteh S, Ntshalintshali N, Nhlabathi N, Dlamini N (2017). Limitations of rapid diagnostic testing in patients with suspected malaria: a diagnostic accuracy evaluation from Swaziland, a low-endemicity country aiming for malaria elimination. Clin Infect Dis.

[32] Hofmann NE, Antunes Moniz C, Holzschuh A, Keitel K, Boillat-Blanco N, Kagoro F (2019). Diagnostic performance of conventional and ultrasensitive rapid diagnostic tests for malaria in febrile outpatients in Tanzania. J Infect Dis.

[33] WHO. Surveillance template protocol for pfhrp2/pfhrp3 gene deletions. Geneva: World Health Organization; 2020. https://www.who.int/publications/i/item/9789240002036.

[34] Grignard L, Nolder D, Sepúlveda N, Berhane A, Mihreteab S, Kaaya R (2020). A novel multiplex qPCR assay for detection of *Plasmodium falciparum* with histidine-rich protein 2 and 3 (pfhrp2 and pfhrp3) deletions in polyclonal infections. EBioMedicine.

