# Supplementary figures and images for: Investigation of Plasmodium falciparum pfhrp2 and pfhrp3 gene deletions and performance of a rapid diagnostic test for identifying asymptomatic malaria infection in northern Ethiopia, 2015
Source: Malar J. 2022 Mar 4;21:70. doi: 10.1186/s12936-022-04097-7 (PMC8895513; doi:10.1186/s12936-022-04097-7)

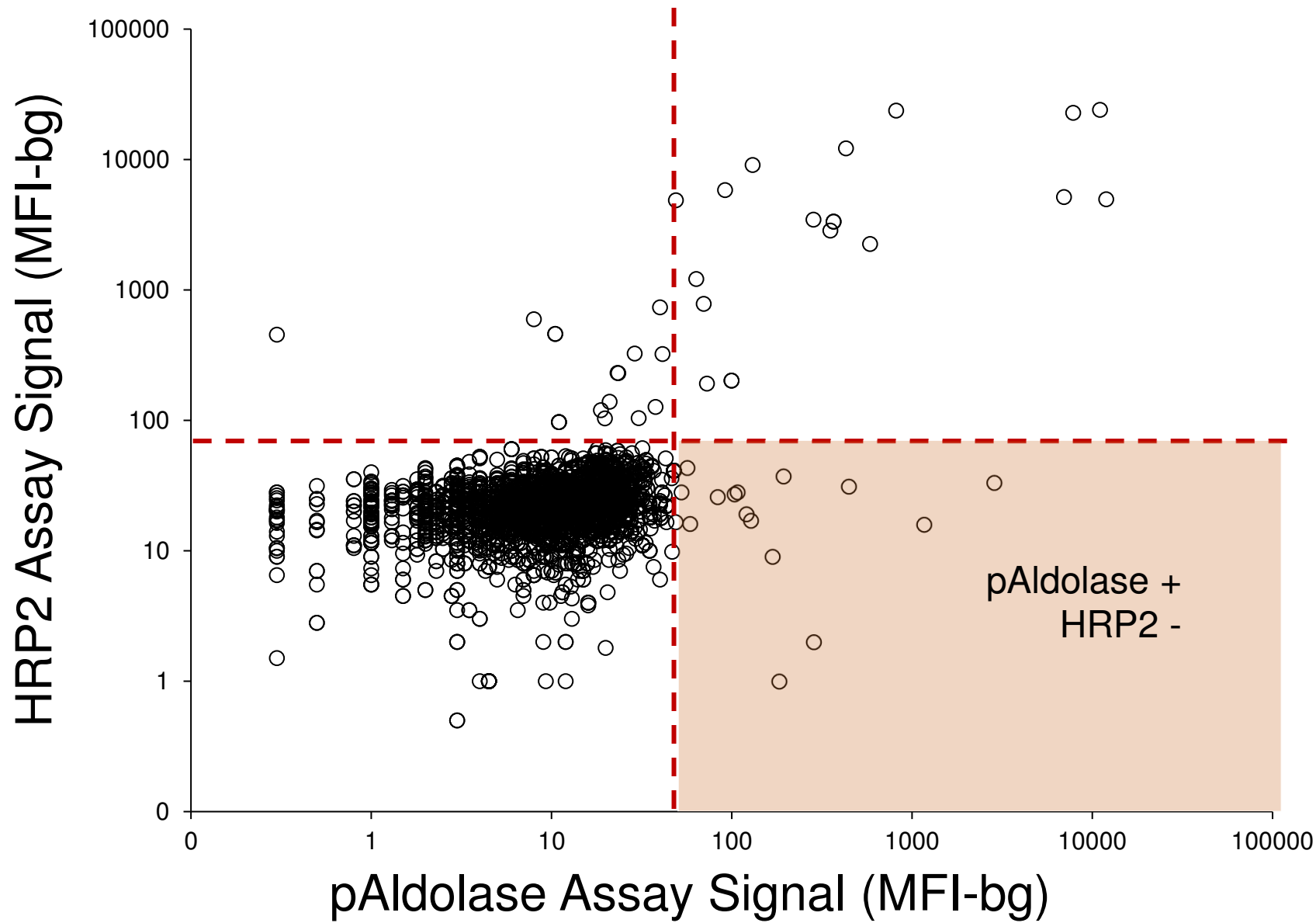

Supplement: Supplementary file 1 — Additional file 1. Selection of samples for pfhrp2 and pfhrp3 genotyping by ratio of pAldolase to HRP2.The vertical hashed red line designates the threshold for antigen positivity signal for pAldolase antigen and the horizontal hashed red line designates the threshold for antigen positivity signal for HRP2. The region shaded in orange denotes values where DBS samples were positive for pAldolase antigen, but negative for HRP2 antigen. [file 12936_2022_4097_MOESM1_ESM.pdf]
